# Dual Light- and pH-Responsive Composite of Polyazo-Derivative Grafted Cellulose Nanocrystals

**DOI:** 10.3390/ma11091725

**Published:** 2018-09-14

**Authors:** Xiaohong Liu, Ming Li, Xuemei Zheng, Elias Retulainen, Shiyu Fu

**Affiliations:** State Key Laboratory of Pulp and Paper Engineering, South China University of Technology, Guangzhou 510640, China; lxh13424067451@163.com (X.L.); li163ming@163.com (M.L.); 13798041124@163.com (X.Z.); Elias.Retulainen@vtt.fi (E.R.)

**Keywords:** cellulose nanocrystals, azo-derivatives, atom transfer radical polymerization, light- and pH-responsive

## Abstract

As a type of functional group, azo-derivatives are commonly used to synthesize responsive materials. Cellulose nanocrystals (CNCs), prepared by acid hydrolysis of cotton, were dewatered and reacted with 2-bromoisobuturyl bromide to form a macro-initiator, which grafted 6-[4-(4-methoxyphenyl-azo) phenoxy] hexyl methacrylate (MMAZO) via atom transfer radical polymerization. The successful grafting was supported by Fourier transform infrared spectroscopy (FT-IR) and Solid magnetic resonance carbon spectrum (MAS ^13^C-NMR). The morphology and surface composition of the poly{6-[4-(4-methoxyphenylazo) phenoxy] hexyl methacrylate} (PMMAZO)-grafted CNCs were confirmed with Transmission electron microscopy (TEM) and X-ray photoelectron spectroscopy (XPS). The grafting rate on the macro-initiator of CNCs was over 870%, and the polydispersities of branched polymers were narrow. The crystal structure of CNCs did not change after grafting, as determined by X-ray diffraction (XRD). The polymer PMMAZO improved the thermal stability of cellulose nanocrystals, as shown by thermogravimetry analysis (TGA). Then the PMMAZO-grafted CNCs were mixed with polyurethane and casted to form a composite film. The film showed a significant light and pH response, which may be suitable for visual acid-alkali measurement and reversible optical storage.

## 1. Introduction

Smart responsive nanomaterials have received much attention over the last decade for their functional properties in response to environmental variables, such as temperature [1,2], humidity [3,4], light [5,6], and pH [7,8]. Developing such smart nanomaterials from widely available natural resources provides an opportunity for high value-added for natural polymers, such as cellulose. Therefore, cellulose nanocrystals (CNCs) are suitable as substrates for these polymers, since they are abundant, renewable, and biocompatible [9,10,11]. CNCs do possess several merits as smart responsive nanomaterials. Chemically, there are many hydroxyl groups on the surface of CNCs, which provide reactive groups for significant surface modification. The high specific surface area of CNCs is also a significant feature, which offers more reaction sites for small-sized particles. Therefore, light-activated or pH-responsive functional groups can be introduced to certain places on the surface of the CNCs [12].

Light is an attractive stimulant, since it does not require direct contact. When light triggering is combined with cellulose nanocrystals, sensitive advanced materials can be applied in the absorption and release of water [13]. Grigoray et al. reported the formation of another photoresponsive cellulose material made by surface modification of fibers with photoactive groups [14]. In addition, the pH value is one of the most critical measurable parameters in many fields, such as medicine, chemistry, biology, and material science. Therefore, the surface modifications of CNCs for pH sensing have also been investigated [15]. However, in this study, we aimed at linking pH- and light-responsive azobenzene derivative groups to CNCs.

Azobenzene moieties have been studied for their acidic discoloration [16], as well as light-induced trans-cis isomerization [17]. Acidic discoloration means a reversible ability to change color with a changing pH environment. Monomers with acidic discoloration provide a reference for applications such as pH measurement or sensors, while the light-induced change, especially a rapid and reversible photoresponsiveness, is a highly desirable material property that can be utilized in smart photoresponsive devices. However, the azobenzene monomer has many drawbacks, such as the poor mechanical properties and processability. Synthesizing the polymers that contain azobenzene side groups can address these problems. Azobenzene-containing polymers (azo polymers) not only possess the azo group of optical activity, but also have excellent mechanical properties and process abilities. Therefore, azo polymer could contribute to a potential use in optical information storage, non-linear optics, liquid crystal material, light regulation, and holographic imaging [18,19,20]. Photoresponsive materials containing azobenzene moieties also have potential applications in micropatterning [21] and actuators [22]. To date, there are a few reports on natural resources-based azo polymers. Tang grafted ethyl cellulose with azobenzene-containing polymethacrylates to make photoresponsive cellulose [23]. Duval et al. reported on the preparation of dual responsive (pH and light) bio-based polymers made by modification of kraft lignin to expose diazobenzene groups [24]. Xu et al. synthetized a novel amphotropic polymer based on cellulose nanocrystals grafted with azo polymers [20]. This is the first report of an azobenzene-containing polymer being grafted from cellulose nanocrystals by the activator regenerated by electron transfer atom transfer radical polymerization (ARGET-ATRP) process. Furthermore, the pH- and light-responsive behaviors of the PMMAZO-grafted CNCs and its composite have been comprehensively studied.

Hypothetically, we have assumed that the combination of cellulose nanocrystals with azobenzene polymer branches should form a smart composite that can change color when exposed to ultraviolet light or changing pH conditions. In this study, PMMAZO-grafted CNCs were synthesized via ARGET-ATRP. The structures of the PMMAZO-graft CNCs were characterized by FT-IR and X-ray diffraction (XRD). The length and density of the grafted polymer were determined by elemental analysis (EA) and gel permeation chromatography (GPC), respectively. The thermal stability was evaluated by thermogravimetry analysis (TGA). Finally, the acidichromatic behavior and light-responsive behavior of the PMMAZO-graft CNCs were also discussed.

## 2. Materials and Methods

### 2.1. Materials

Cotton linters with a moisture content of 8% and α-cellulose content more than 98% were supplied by Fumin Chemical Fiber Co. Ltd. (Shandong, China). The shape-memory polyurethane elastomer (SMP) was purchased from Lixin Material Manufacture Co. Ltd. (Guangdong, China). Sulfuric acid (98 wt%), *N*,*N*,*N*′,*N*″,*N*″-pentamethyldiethylenetriamine (PMDETA, Aldrich, 98%) was used as received. 2-Bromoisobuturyl bromide (BIBB) (98%, Alfa, Aesar), triethylamine (TEA, 99%), 4-dimethylaminopyridine (DMAP, 99%), and L-ascorbic acid (AsAc, 99%) were purchased from Aladdin Chemical Co. Ltd. (Shanghai, China). TEA was refluxed with tosyl chloride for 12 h and then distilled at 89.5 °C. The distillate was dried to remove the primary amines and secondary amines. Cu(I)Br (from Aldrich) was purified in three steps: stirring in glacial acetic acid, washing with ethanol three times, and then drying the Cu(I)Br in vacuum at room temperature overnight. 1-Methyl-2-pyrrolidinone (NMP, Aldrich, Shanghai, China) and *N*,*N*-dimethylformamide (DMF, Aldrich, Shanghai, China) was dried with a molecular sieve over three days. The MMAZO was purchased from Shanghai SummerPharm Tech Co. Ltd., Shanghai, China. All solvents were purified before use.

### 2.2. Methods

#### 2.2.1. Preparation of Cellulose Nanocrystals

Cellulose nanocrystals were prepared according to the method of our group [25]. The cotton linters were hydrolyzed with H_2_SO_4_ (64 wt%) with ratios of acid to cotton linters of 8.75–17.5 mL∙g^−1^ and hydrolysis proceeds at 45 °C for 1.5 h under vigorous mechanical stirring. The obtained suspension was diluted by deionized water and centrifuged until no stratification was present. Then it was dialyzed against deionized water using dialysis tubing with an 8000–14,000 molecular weight cut-off (MWCO) until neutral. Finally, the suspension was ultrasonicated at room temperature to obtain a uniform CNC dispersion. CNC powder was recovered by freeze-drying prior to use.

#### 2.2.2. Immobilization of Initiator on Cellulose Nanocrystals

The procedure followed here is the same as executed in [26]. Dried CNCs (9.72 g) and 300 mL of NMP solvent were introduced into a flask and treated with an ultrasonic device for 5 min, and then stirred overnight. A total of 250 mL of NMP solvent, 0.090 mol of TEA, and 0.050 mol of DMAP were added to the above flask with degassing and nitrogen protection in an orderly fashion. NMP solution (50 mL) containing 0.36 mol of BIBB was then added dropwise into the flask to initiate the reaction. The reaction proceeded under nitrogen at 0 °C for 2 h and then at room temperature for 24 h. The crude product was centrifuged with ethanol and water, respectively. It was then extracted with EtOH/H_2_O and then dispersed in water and recovered by freeze-drying. The end product (CNCs-IBBr) was vacuum-dried at 45 °C until it reached a constant weight (10.1 g). Since weight loss occurred during washing, the actual weight of the CNCs-IBBr (M) was more than 10.1 g.

The analytical results are as follows: The actual weight of the CNCs-IBBr (M), Degree of substitution (DS) and substitution were calculated according to Equation (1):

$$M\times 8.88\%/M_{Br}\times M_{IBBr}=M-9.72$$

$$DS=n_{Br}/n_{AGU}$$

(1) $$\mathrm{substitution}=\frac{1}{3}DS\times 100\%$$

where *M_Br_* and *M_IBBr_* are the molecular masses of Br and IBBr, respectively. *n_Br_* and *n_AGU_* are the amount of Br and anhydroglucose.

Oxygen bomb ion chromatography: Br, 8.88 wt%. *M* was 11.7 g. Average degree of substitution (*DS*, defined as the number of reacted hydroxyl groups in one unit of cellulose fiber) at the esterification step = 0.22 (7.3% substitution) [27].

#### 2.2.3. Grafting of MMAZO from Initiator-Functionalized Cellulose Nanocrystals

The CNCs-IBBr was used to initiate the polymerization of MMAZO via ATRP using CuBr/PMDETA as a catalyst system [28]. In a general procedure, CNCs-IBBr (0.300 g, 0.150 mmol of Br), MMAZO (8.00 g, 22.2 mmol), PMDETA (0.0693 g, 0.400 mmol), AsAc (350 mg, 0.200 mmol) and DMF (15.0 mL) were added into a dry flask with a magnetic stirring bar. After the macro-initiator was dispersed evenly, Cu(I)Br (28.8 mg, 0.200 mmol) was added into the flask, and then the reaction system was degassed with three freeze-evacuate-thaw cycles. Thereafter, the flask was immersed into an oil bath at 60 °C. The reaction was stopped by exposing the mixture to air and diluting with ethanol. The crude product was centrifuged with ethanol and water, respectively. It was extracted with EtOH/H_2_O for 24 h, then recovered by freeze-drying. The end product (PMMAZO-grafted CNCs) was vacuum-dried at 45 °C for 48 h to reach a constant weight.

To estimate the graft ratio, the CNCs-IBBr and PMMAZO-grafted CNCs samples were weighed. The graft ratio (G, wt%) was calculated using Equation (2) [29]:

(2) $$G=\left(W_{2}-W_{1}\right)/W_{1}\times 100\%$$

where *W*_1_ (0.300 g) is the dry weight of the CNCs-IBBr sample and *W*_2_ (2.90 g) is the dry weight of the PMMAZO-grafted CNCs sample. Based on these, the graft ratio in this article was 870%.

#### 2.2.4. Preparation of PMMAZO-Grafted CNCs/PU Films

PMMAZO-grafted CNCs were firstly dispersed in DMF via an ultrasonic treatment (under this condition, the suspension can remain stable for a long time). Thereafter, the polyurethane was dissolved in DMF, then mixed with the above-mentioned PMMAZO-grafted CNCs suspension through magnetic stirring for 6 h. The resulting mixtures were subsequently cast into circular polytetrafluoroethylene (PTFE) molds (6 cm in diameter) and oven-dried at 80 °C for 8 h.

#### 2.2.5. Cleavage of Polymer Brushes from PMMAZO-grafted CNCs

A total of 0.1 g of cellulose nanocrystal-based copolymer (PMMAZO-grafted CNCs) was added into a mixture solution of 37% HCl (20 mL) and THF (50 mL), then refluxed at 90 °C for three weeks to dissolve the PMMAZO-grafted CNCs completely. Subsequently, the red solution mixture was filtered and evaporated by a rotary evaporator. Then it was directly vacuum-dried, and the resulting yellow product was dissolved into tetrahydrofuran (THF) and filtered through a 0.45 mm PET syringe filter prior to GPC analysis [27]. The process of cleaving PMMAZO from CNCs was the process of hydrolyzing the CNCs in the PMMAZO-grafted CNCs with acid so that the PMMAZO can dissolve in the THF. In the process, it was difficult to purity the PMMAZO that cleaved from the PMMAZO from CNCs, since it may include some hydrolysate of CNCs. This may not affect the determination of molecular weight, but it may be inappropriate to run an ^1^H Nuclear Magnetic Resonance Spectra (^1^H-NMR) and ^13^C Nuclear Magnetic Resonance Spectra (^13^C-NMR).

#### 2.2.6. Measurement of the Responsive Behavior of PMMAZO-Grafted CNCs and Their Composite

To study the pH responsiveness of PMMAZO-grafted CNCs solutions, 25 µL stock solutions of the PMMAZO-grafted CNCs in ethanol (4 g^−1^) were diluted to 2 mL with deionized water previously adjusted to pH 1 to pH 5, and all of them can form stable suspension and the spectra of MMAZO when different pH were recorded under the same conditions. A total of 4 mL of stock solution of MMAZO in ethanol (3 × 10^−5^ mol/L) was tested by means of exposure to UV light (365 nm). Then, its reversible spectra were also measured after removal of the UV light. The photochromism of PMMAZO-grafted CNCs was measured by exposing the composite to the UV light (365 nm), and its reversibility was also studied by dark reaction.

#### 2.2.7. TEM, FT-IR, NMR, XPS, and EA Analysis

Transmission electron microscopy (TEM), Fourier transform infrared spectroscopy (FT-IR), nuclear magnetic resonance (NMR), and X-ray photoelectron spectroscopy (XPS) were used to determine the morphologies and sizes of CNCs, the structural characteristics, and elemental atomic compositions in CNCs, CNCs-IBBr, and PMMAZO-grafted CNCs, respectively. TEM was recorded on an H-7650 (Hitachi, Japan) at 80 kV accelerating voltage. The nanocellulose samples stained by 3 wt% solution of phosphotungstic acid were prepared for TEM. FT-IR spectra were recorded on a Bruker TENSOR27 Spectrum (Bruker Corporation, Karlsruhe, Germany) FT-IR spectrophotometer, and the samples were prepared by dispersing the complexes in KBr and compressing the mixtures to form disks, as well as being recorded in the range of 4000–400 cm^−1^ for each spectrum. MAS ^13^C NMR was recorded on a Bruker AV-III 400 MHz spectrometer (Bruker Corporation, Karlsruhe, Germany). Oxygen bomb ion chromatography was used to measure the bromine content. XPS measurements were obtained on an ESCA Lab MK II (V.G. Scientific Co. Ltd., Bristol, UK) equipped with an Mg KR radiation source (12 kV and 20 mA at the anode). The take-off angle of the photoelectron was kept at 45°. The binding energy was referenced by setting the C1s hydrocarbon peak at 285.0 eV. Elemental analysis (EA, Elementar, Hanau, Germany) was used to calculate the DS of the CNCs-IBBr.

#### 2.2.8. XRD, TGA, UV–VIS Absorption Spectra and GPC

X-ray diffraction (XRD), thermogravimetry analysis (TGA) and UV–VIS absorption spectra were used to characterize the crystalline structure, decomposition behavior, pH- and light-responsiveness of CNCs, CNCs-IBBr, and PMMAZO-grafted CNCs, respectively. XRD measurements were performed on a D8 ADVANCE wide angle X-ray diffractometer system (Bruker Corporation, Karlsruhe, Germany). The diffracted intensity of Cu Kα radiation (k = 0.1542 nm; 40 kV and 40 mA) was measured in a 2θ range between 5° and 60°. TGA was made using a seven-series thermal analysis system (PerkinElmer, Waltham, MA, USA). Samples were heated from room temperature to 600 °C at 10 °C/min in a dynamic nitrogen atmosphere at a flow rate of 25 mL/min. The UV–VIS absorption spectra were recorded using an HP-8453 UV–VIS spectrometer (Agilent, CA, USA). The number-average molecular weight (Mn) and molecular weight distribution (MWD) of the polymer cleaved from PMMAZO-grafted CNCs were measured on a GPCMax VE201 gel permeation chromatography (GPC) (Viscotek Co., Houston, TX, USA) equipped with three columns PAA-202-204-205 and three detectors: a viscometer, light scattering, and IR detector. The calibration was made with poly (ethylene oxide) standards. 

## 3. Results and Discussion

### 3.1. Cellulose Nanocrystals Grafted with PMMAZO

**Scheme 1:**
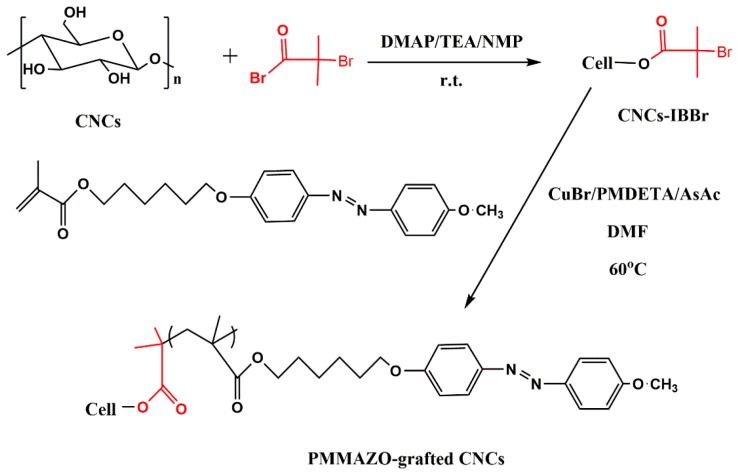
Illustration of the functionalization of the cellulose nanocrystals. In the first step, the hydroxyl groups on the CNCs were functionalized with BIBB. In the second step, MMAZO was grafted from the surface of the CNCs via ARGET-ATRP.

To generate the functional PMMAZO-graft CNCs, we use ARGET-ATRP method, which is particularly useful for the preparation of designed polymers and materials with complex architectures [30]. With this method, we can graft polymer chains with the monomer contained azo-group on CNCs. The procedure of reactions is shown in Scheme 1.

**Figure 1:**
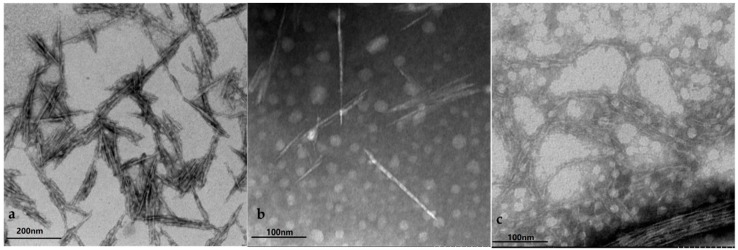
TEM images of CNCs (**a**); CNCs-IBBr (**b**); PMMAZO-grafted CNCs (**c**).

The morphology of CNCs, CNCs-IBBr and PMMAZO-grafted CNCs are depicted in Figure 1 by TEM observation. The CNCs shown in Figure 1a were rod-like particles with lengths of 150–250 nm and widths of 10–20 nm (giving an aspect ratio of 10–12) that were well dispersed with few aggregates. The morphology of the CNCs-IBBr shown in Figure 1b was just like that of the CNCs, while the PMMAZO-grafted CNCs shown in Figure 1c became coarse and wide, causing a rigid rod-like structure not as clear as the pure CNCs. The change of the morphological integrity before and after grafting modification could indicate that the polymer brushes was successfully grafted from CNCs. This is the same conclusion arrived at by other research [27].

**Figure 2:**
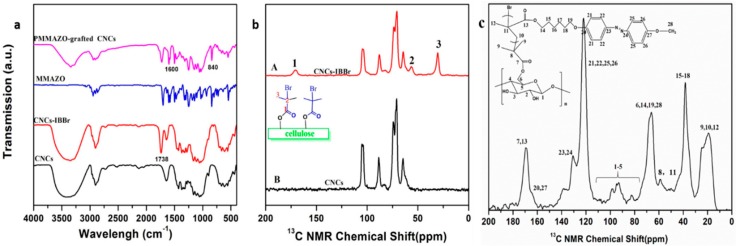
FT-IR structural characteristics of: (**a**) pure CNCs, CNCs-IBBr, MMAZO, and PMMAZO-grafted CNCs; (**b**) MAS 13C NMR spectrum of pure CNCs and CNCs-IBBr; and (**c**) MAS 13C NMR spectrum of PMMAZO-grafted CNCs.

The FT-IR spectra of the pure CNCs, CNCs-IBBr, MMAZO, and PMMAZO-grafted CNCs are shown in Figure 2a. A small carbonyl peak (1738 cm^−1^) could be observed in the spectra of initiator-modified CNCs that was attributed to the carbonyl group in the initiator, which was fixed on the CNCs surface [29]. For the PMMAZO-grafted CNCs, an additional strong absorption band shows at 1600 cm^−1^, which was attributed to the stretching vibration of the aromatic rings in MMAZO. Furthermore, the peak of the PMMAZO-grafted CNCs samples around 840 cm^−1^ belonged to the two adjacent hydrogen atoms on the benzene ring. These results confirmed that PMMAZO was immobilized on the CNCs surface. The MAS ^13^C NMR spectra of pure CNCs, CNCs-IBBr are shown in Figure 2b. There was a typical signal of carbon atoms of cellulose nanocrystals backbone in unmodified CNCs (Curve A), and no other signal was observed. However, several new chemical shifts of carbon atoms appeared at 172 ppm, 56 ppm, and 30 ppm in the initiator-modified CNCs (Curve B), which was attributed to 1, 2, and 3, respectively [31]. The MAS ^13^C NMR spectrum of the PMMAZO-grafted CNCs (Figure 2c) showed the typical signals of MMAZO at 169.5, 130.6, 121.9, 66.3, 38.2 ppm, besides the signals at 59.0–107 ppm and 170 ppm from CNCs-IBBr. Compared with Figure 2b, the signal belonging to the carbons of the methyl of isobutyryl bromide group was shifted to the high field (at 18.9 ppm). The phenomenon could be explained by the liberation of bromide atoms with high electronegativity from the carbon being adjacent to the methyl carbon.

**Figure 3:**
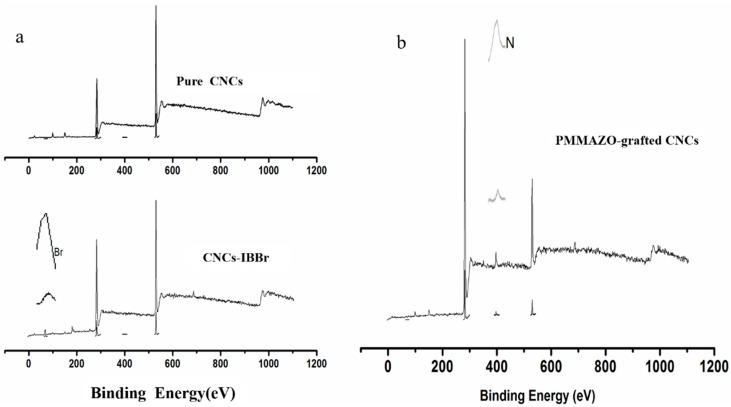
The low-resolution map of pure CNCs, CNCs-IBBr (**a**), and PMMAZO-grafted CNCs (**b**).

**Figure 4:**
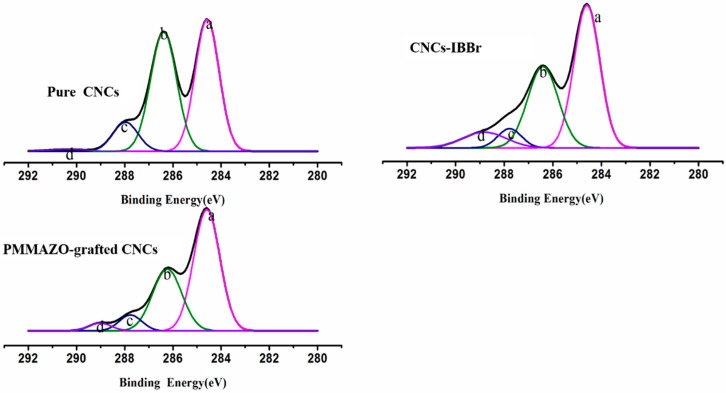
The high-resolution C1s spectra of pure CNCs, CNCs-IBBr and PMMAZO-grafted CNCs.

The surface composition of the pure CNCs, CNCs-IBBr, and PMMAZO-grafted CNCs was characterized by XPS. There was a peak at 68 eV (Figure 3a), which corresponds to the element bromine. The measurement showed that the CNCs-IBBr contained 6.27% of the bromine element, while the pure CNC did not, which indicated that BIBB group was grafted on CNCs by esterification of surface hydroxyl group. Furthermore, when we prepared the PMMAZO-grafted CNCs, we also tested the nitrogen components on the surface of products and found an approximate content of 6.15% nitrogen (Figure 3b) was detected at 402.5 eV. To obtain more detailed information about the grafted group, the high-resolution C1s spectrum analysis was adopted (Figure 4). For the high-resolution C1s spectrum, the carbon atom signal can be divided into four different Gaussian peaks [32]: the peak at 285.0 eV represents C–C or C–H bonding (a), the peak at 286.5 eV represents C–O bonding (b), the peak at 288.0 eV represents O–C–O bonding (c), and the peak at 289.0 eV represents O–C=O bonding (d). For the CNCs-IBBr, the peak at 289 eV(d) increased 8.079% after immobilization of the initiator on cellulose nanocrystals, which demonstrated that there were more C=O on the initiator’s immobilized surface. Furthermore, when the polymerization lasted for 24 h, the signal of the C=O declined 6.591% again, suggesting that the grafted PMMAZO layer covered the surface of CNCs-IBBr. In addition, MMAZO also had C=O group, but its relative content was low, so the signal for C=O in the end product was weak [33].

**Figure 5:**
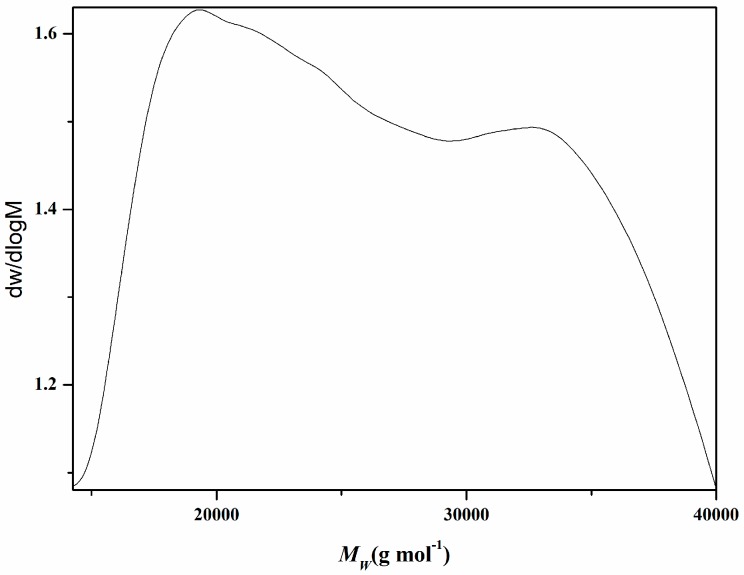
The molecular weight distribution of PMMAZO which cleaved from the PMMAZO-grafted CNCs.

Table 1 shows the monomer conversion, molecular weights, and polydispersities of grafted polymers during ATRP. The monomer conversion was 32.5%, which was calculated according to Equation (3). Taking into account the weight loss during washing, the actually monomer conversion should be slightly higher. To further confirm the characteristics of PMMAZO chains grafted from CNCs by ATRP, the PMMAZO was cleaved from the PMMAZO-grafted CNCs via hydrolysis. The molecular weight of the cleaved polymers from the GPC analysis was 18791, the theoretical average molecular weight was 15533, which was calculated according to Equations (4) and (5). the grafted polymers of surface-grafted CNCs revealed moderate polydispersity indices (PDI, ~1.27), which showed that the ATRP polymerization was a controlled process. As shown in Figure 5, the GPC results indicated bi-model peaks and broad molecular weight distribution. The second weak peak suggested that a small portion of PMMAZO had relatively high molecular weight.

(3) $$\mathrm{monomer}\mathrm{conversion}\left(\%\right)=\left(w_{2}-w_{1}\right)/w_{1}\times 100\%$$

(4) $$C_{Br}=\frac{3}{2}\times \frac{9.72}{M\times n_{AGU}}\times \mathrm{substitution}$$

(5) $$M_{n,\mathrm{theroretical}}=\frac{{∆}_{m}}{C_{Br}\times m_{0}}+M_{initiator}$$

where *W*_1_ (8.00 g) was the dry weight of the MMAZO which was added into the flask initially and *W*_2_ (2.60 g) was the dry weight of the MMAZO grafted from the CNCs. *M* was the actual weight of the CNCs-IBBr, ${∆}_{m}$ was the increased weight of CNCs after grafting. $C_{Br}$ was the bromine content of CNCs-IBBr. *m*_0_ is the initial mass of CNCs-IBBr; $M_{initiator}$ is the molecular weight of immobilized initiator on CNCs.

### 3.2. Properties of the PMMAZO-Grafted CNCs

#### 3.2.1. Crystal Structure and Thermal Analysis

**Figure 6:**
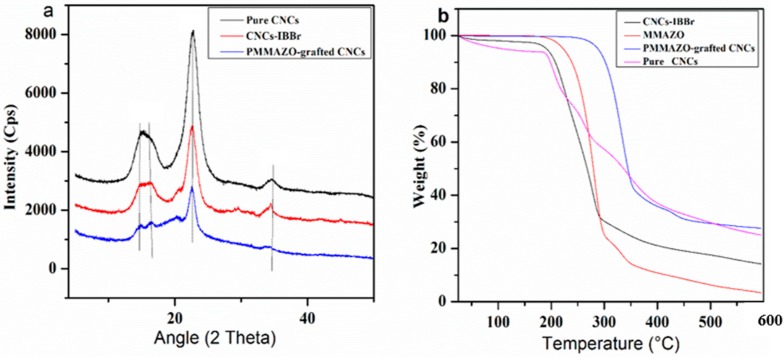
XRD pattern (**a**) of pure CNCs, CNCs-IBBr and PMMAZO-grafted CNCs; and (**b**) the TG curves of pure CNCs, CNCs-IBBr, and PMMAZO-grafted CNCs.

The crystal structure of cellulose nanocrystals and its modified products were analyzed by XRD. The XRD patterns are shown in Figure 6a. The results of calculating the corresponding crystallinity are shown in Table 2. As can be seen from Figure 6a, the pure CNCs had a typical cellulose I crystal structure, which had typical peaks at 14.6°, 16.2°, 22.6°, and 34.0° [34,35]. The CNCs-IBBr and PMMAZO-grafted CNCs still maintained the diffraction peaks of the major crystal faces despite being modified or grafted, which indicated that the crystal structure of CNCs did not change during grafting. However, the crystallinity of CNCs-IBBr and PMMAZO-grafted CNCs became lower than that of pure CNCs, i.e., the crystallization zone of cellulose nanocrystals was lost to a certain extent during grafting. Furthermore, the hydroxyl groups on the surface of cellulose were replaced by 2-bromoisobutyryl bromide, which weakened the hydrogen bonding between cellulose nanocrystals [36].

Figure 6b shows thermogravimetric curves for pure CNCs, CNCs-IBBr, pure MMAZO, and PMMAZO-grafted CNCs. For pure CNCs and CNCs-IBBr, there was some weight loss in the low temperature range (<120 °C), corresponding to the evaporation of absorbed water [37]. Pure MMAZO decomposed without residual mass, while the pure CNCs yielded a higher amount of residue (25 wt%). The CNCs prepared by sulfuric acid hydrolysis showed a typical degradation behavior with an onset temperature of about 250 °C. This value was relatively lower than that of the neutral, sulfate-free cellulose; the difference came from the sulfonic acid group remaining in CNCs, which induced thermal degradation at lower temperatures [38]. When CNCs were reacted with BIBB to form the CNCs, thermal stability decreased. This may be attributable to the labile bromine within the CNCs-IBBr, which could speed up the decomposition of CNCs with free radical mechanisms [29]. On the other hand, the decomposition temperature of PMMAZO-grafted CNCs changed to 318 °C, which was higher than pure CNCs. The reason is that the side chain polymer MMAZO may improve the thermal stability of cellulose nanocrystals [20].

#### 3.2.2. pH-Responsive Properties of the PMMAZO-Grafted CNCs

**Figure 7:**
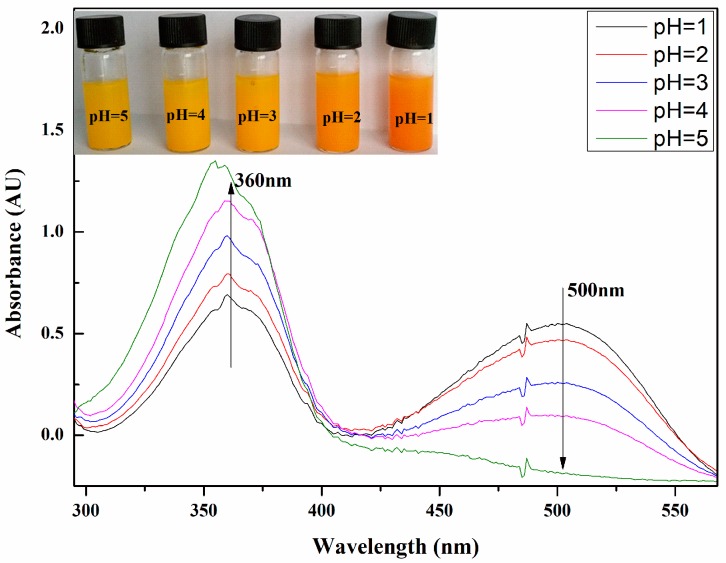
PMMAZO-grafted CNCs aqueous solutions (c = 0.2 g·L^−1^) at different pH, UV–VIS spectra of the MMAZO solution at different pH.

The MMAZO grafted from CNCs is widely known for its pH-sensitivity. It happens to have the same structure as diazobenzene [24], solution of which undergoes a red to yellow color change when pH increases above 3. Therefore, the pH-response of PMMAZO-grafted CNCs samples was tested by means of UV–VIS spectra (Figure 7). The PMMAZO-grafted CNCs dissolved in ethanol to form solutions, then the solutions were diluted with water previously adjusted to a pH within the range of 1 to 5. The picture in Figure 7 shows the color changed from red to yellow when the pH changed from 2 to 3. This was also confirmed by the UV–VIS spectra, which caused an important decrease of the peak located at 500 nm and an increase of the absorption around 360 nm [24].

#### 3.2.3. Light-Responsive Behavior

**Figure 8:**
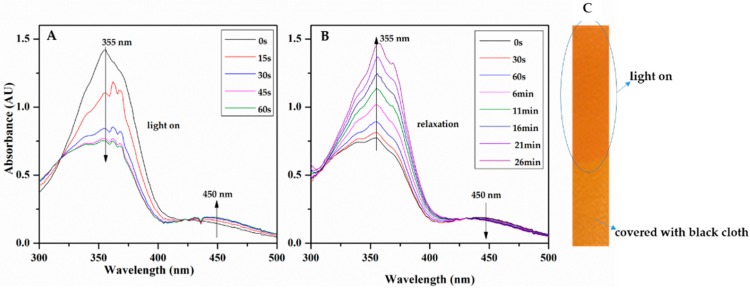
The light-responsive behavior of the MMAZO in ethanol (**A** and **B**) and the PMMAZO-graft CNCs and polyurethane composite film (**C**).

The light-responsive behavior of the MMAZO in ethanol was studied by the means of UV (Figure 8). The results indicate that the ultraviolet spectrum of MMAZO solution changed significantly when exposed to the UV light (365 nm) at different times (the time of each line was 15 s) (Figure 8A), which caused a strong reduction in the π−π* absorption (355 nm) band and a slight increase in the n−π* absorption (450 nm) band. The result should be related to the transition from the trans to the cis [39]. Reverse isomerization was also demonstrated from the absorption spectra (Figure 8B). Oppositely, the absorption stemmed from the π−π* transition band (355 nm) increased obviously upon irradiation with natural light, whereas the n−π* absorption (450 nm) band weakened. Finally, the absorption peak was restored to its original state [40]. For a more intuitive observation, polyurethane composite film was made by mixing the PMMAZO-graft CNCs and polyurethane (the PMMAZO-graft CNCs content in component is 20%). As shown in Figure 8a, part of the film covered with black cloth did not change color, while the other part exposed to ultraviolet light (light on) became red. The color changes originated from the trans-cis photosiomerization of the MMAZO grafted cellulose nanocrystals, which indicated that the MMAZO had grafted on the CNCs successfully.

## 4. Conclusions

We explored cellulose nanocrystals functionalized with poly{6-[4-(4-methoxyphenylazo) phenoxy] hexyl methacrylate} (PMMAZO) that could change color when exposed to ultraviolet light or changing pH conditions. This behavior was reversible, which was attributable to the cis-trans photoisomerization of the azobenzene derivative directly grafted on the CNCs. Overall, the results support the hypothesis that the combination of cellulose nanocrystals with azobenzene polymers should change color when exposed to ultraviolet light or changing pH conditions. There was no observable damage to the crystalline structure of CNCs due to grafting. The side chain of PMMAZO can improve the thermal stability of the cellulose nanocrystals by a polymer shield. In addition, the prepared cellulose nanocrystals derivatives (PMMAZO-grafted CNCs) could disperse well in apolar organic media (chloroform solvents and PU polymer matrices). The PMMAZO-grafted CNCs/PU films demonstrated visually pH- and light-responsive behavior. Overall, this work has provided an appropriate way to make nanocellulose-derived stimuli-responsive polymers. The results indicate that CNCs can be converted into novel multifunctional nanomaterials, which would open up possible applications for visual-acid-alkali measurement and reversible optical storage. In order to determine the full potential of the described functionalized cellulose, more detailed studies of these pH- and light-responsive properties, including the homogeneous ATRP, are currently being researched in our laboratory.

## Figures and Tables

**Table 1:** Conversion, molecular weights, and polydispersities of grafted polymers during ATRP.

Sample	Conversion	Mn	PDI
PMMAZO-grafted CNCs	32.5%	18,791	1.27

**Table 2:** The degree of crystallinity for Pure CNCs, CNCs-IBBr, and PMMAZO-grafted CNCs.

Sample	Degree of Crystallinity (%)
Pure CNCs	76.21%
CNCs-IBBr	66.49%
PMMAZO-grafted CNCs	48.41%

## References

[1] Yang H., Esteves A.C.C., Zhu H., Wang D., Xin J.H. (2012). In-situ study of the structure and dynamics of thermo-responsive PNIPAAm grafted on a cotton fabric. Polymer.

[2] Zoppe J.O., Habibi Y., Rojas O.J., Venditti R.A., Johansson L.S., Efimenko K., Osterberg M., Laine J. (2010). Poly(*N*-isopropylacrylamide) brushes grafted from cellulose nanocrystals via surface-initiated single-electron transfer living radical polymerization. Biomacromolecules.

[3] Yang H., Zhu H., Hendrix M.M., Lousberg N.J., De With G., Esteves A.C., Xin J.H. (2013). Temperature-triggered collection and release of water from fogs by a sponge-like cotton fabric. Adv. Mater..

[4] Yu H.W., Kim H.K., Kim T., Bae K.M., Seo S.M., Kim J.M., Kang T.J., Yong H.K. (2014). Self-Powered Humidity Sensor Based on Graphene Oxide Composite Film Intercalated by Poly(Sodium 4-Styrenesulfonate). ACS Appl. Mater. Interfaces.

[5] Klajn R. (2014). Spiropyran-based dynamic materials. Chem. Soc. Rev..

[6] Ueki T., Usui R., Kitazawa Y., Lodge T.P., Watanabe M. (2015). Thermally Reversible Ion Gels with Photohealing Properties Based on Triblock Copolymer Self-Assembly. Macromolecules.

[7] Way A.E., Hsu L., Shanmuganathan K., Weder C., Rowan S.J. (2012). pH-Responsive Cellulose Nanocrystal Gels and Nanocomposites. ACS Macro. Lett..

[8] Li Z., Shen J., Ma H., Lu X., Shi M., Li N., Ye M. (2013). Preparation and characterization of pH- and temperature-responsive nanocomposite double network hydrogels. Mater. Sci. Eng. C Mater..

[9] Hu J., Meng H., Li G., Ibekwe S.I. (2012). A review of stimuli-responsive polymers for smart textile applications. Smart Mater. Struct..

[10] Bashari A., Nejad N.H., Pourjavadi A. (2013). Applications of stimuli responsive hydrogels: A textile engineering approach. J. Text. Inst. Proc. Abstr..

[11] Ganesh V.A., Baji A., Ramakrishna S. (2014). Smart functional polymers—A new route towards creating a sustainable environment. Rsc Adv..

[12] Habibi Y. (2014). Key advances in the chemical modification of nanocelluloses. Chem. Soc. Rev..

[13] Schiphorst J.T., Broek M.V.D., Koning T.D., Murphy J.N., Schenning A.P.H.J., Esteves A.C.C. (2016). Dual light and temperature responsive cotton fabric functionalized with a surface-grafted spiropyran–NIPAAm-hydrogel. J. Mater. Chem. A.

[14] Grigoray O., Wondraczek H., Heikkilä E., Fardim P., Heinze T. (2014). Photoresponsive cellulose fibers by surface modification with multifunctional cellulose derivatives. Carbohyd. Polym..

[15] Ma Q., Wang L. (2016). Preparation of a visual pH-sensing film based on tara gum incorporating cellulose and extracts from grape skins. Sens. Actuators. B Chem..

[16] Yuan T., Dong J., Han G., Wang G. (2016). Polymer nanoparticles self-assembled from photo-, pH- and thermo-responsive azobenzene-functionalized PDMAEMA. Rsc. Adv..

[17] Yu H., Kobayashi T. (2010). Photoresponsive block copolymers containing azobenzenes and other chromophores. Molecules.

[18] Berberova N., Daskalova D., Strijkova V., Kostadinova D., Nazarova D., Nedelchev L., Stoykova E., Marinova V., Chi C.H., Lin S.H. (2017). Polarization holographic recording in thin films of pure azopolymer and azopolymer based hybrid materials. Opt. Mater..

[19] Meng X., Natansohn A., Rochon P. (2015). Azo polymers for reversible optical storage. 11 poly{4,4′-(1-methylethylidene)bisphenylene 3-[4-(4-nitrophenylazo)phenyl]-3-aza-pentanedioate}. J. Polym. Sci. Phys..

[20] Xu Q., Yi J., Zhang X., Zhang H. (2008). A novel amphotropic polymer based on cellulose nanocrystals grafted with azo polymers. Eur. Polym. J..

[21] Sobolewska A., Bartkiewicz S., Miniewicz A., Schabbalcerzak E. (2010). Polarization dependence of holographic grating recording in azobenzene-functionalized polymers monitored by visible and infrared light. J. Phys. Chem. B.

[22] Li M.H., Keller P., Li B., Wang X., Brunet M. (2003). Light-Driven Side-On Nematic Elastomer Actuators. Adv. Mater..

[23] Tang X., Gao L., Fan X., Zhou Q. (2010). Controlled grafting of ethyl cellulose with azobenzene-containing polymethacrylates via atom transfer radical polymerization. J. Polym. Sci. Pol. Chem..

[24] Duval A., Lange H., Lawoko M., Crestini C. (2015). Modification of Kraft Lignin to Expose Diazobenzene Groups: Toward pH- and Light-Responsive Biobased Polymers. Biomacromolecules.

[25] Hu Z., Meng Q., Liu R., Fu S., Lucia L.A. (2017). Physical Study of the Primary and Secondary Photothermal Events in Gold/Cellulose Nanocrystals (AuNP/CNC) Nanocomposites Embedded in PVA Matrices. ACS Sustain. Chem..

[26] Li M., Liu X., Liu N., Guo Z., Singh P.K., Fu S. (2018). Effect of surface wettability on the antibacterial activity of nanocellulose-based material with quaternary ammonium groups. Colloids Surf. A.

[27] Chen J., Yi J., Sun P., Liu Z.T., Liu Z.W. (2009). Grafting from ramie fiber with poly(MMA) or poly(MA) via reversible addition-fragmentation chain transfer polymerization. Cellulose.

[28] Xiao M., Li S., Chanklin W., Zheng A., Xiao H. (2011). Surface-initiated atom transfer radical polymerization of butyl acrylate on cellulose microfibrils. Carbohyd. Polym..

[29] Sui X., Yuan J., Zhou M., Zhang J., Yang H., Yuan W., Wei Y., Pan C. (2008). Synthesis of cellulose-graft-poly(*N*,*N*-dimethylamino-2-ethyl methacrylate) copolymers via homogeneous ATRP and their aggregates in aqueous media. Biomacromolecules.

[30] Dong X., Bao H., Ou K., Yao J., Zhang W., He J. (2015). Polymer-grafted modification of cotton fabrics by SI-ARGET ATRP. Fibers Polym..

[31] Meng T., Gao X., Zhang J., Yuan J., Zhang Y., He J. (2009). Graft copolymers prepared by atom transfer radical polymerization (ATRP) from cellulose. Polymer.

[32] Labet M., Thielemans W. (2011). Improving the reproducibility of chemical reactions on the surface of cellulose nanocrystals: ROP of ε-caprolactone as a case study. Cellulose.

[33] Liu P.S., Chen Q., Liu X., Yuan B., Wu S.S., Shen J., Lin S.C. (2009). Grafting of zwitterion from cellulose membranes via ATRP for improving blood compatibility. Biomacromolecules.

[34] Hu D., Wang L. (2016). Preparation and characterization of antibacterial films based on polyvinyl alcohol/quaternized cellulose. React. Funct. Polym..

[35] Cheng B., Ren Y., Wang Y., Ding C. (2007). Synthesis and characterization of cellulose carbamate. J. Text. Res..

[36] Liu X., Chen J., Sun P., Liu Z.W., Liu Z.T. (2010). Grafting modification of ramie fibers with poly(2,2,2-trifluoroethyl methacrylate) via reversible addition–fragmentation chain transfer (RAFT) polymerization in supercritical carbon dioxide. React. Funct. Polym..

[37] Fernándezquiroz D., Gonzálezgómez Á., Lizardimendoza J., Vázquezlasa B., Goycoolea F.M., San R.J., Argüellesmonal W.M. (2015). Effect of the molecular architecture on the thermosensitive properties of chitosan-g-poly(*N*-vinylcaprolactam). Carbohyd. Polym..

[38] Roman M., Winter W.T. (2004). Effect of sulfate groups from sulfuric acid hydrolysis on the thermal degradation behavior of bacterial cellulose. Biomacromolecules.

[39] Yoshida T., Kanaoka S., Aoshima S. (2010). Photo-responsive copolymers with azobenzene side groups synthesized by living cationic polymerization: Efficient amplification of photosensitivity in aqueous photo-switching system. J. Polym. Sci. Pol. Chem..

[40] Bandara H.M., Burdette S.C. (2012). Photoisomerization in different classes of azobenzene. Chem. Soc. Rev..

